# The Intracellular Growth of *M. tuberculosis* Is More Associated with High Glucose Levels Than with Impaired Responses of Monocytes from T2D Patients

**DOI:** 10.1155/2019/1462098

**Published:** 2019-11-14

**Authors:** Martha Torres, María Teresa Herrera, Guadalupe Fabián-San-Miguel, Yolanda Gonzalez

**Affiliations:** ^1^Subdirección de Investigación Biomédica, National Institute of Respiratory Diseases, Mexico City, Mexico; ^2^Department for Microbiology Research, National Institute of Respiratory Diseases, Mexico City, Mexico; ^3^Clinic for Metabolic Syndrome, National Institute of Respiratory Diseases, Mexico City, Mexico

## Abstract

Diabetes mellitus, a metabolic disease characterized by hyperglycemia and poor glucose control, is a risk factor for *Mycobacterium tuberculosis* (*M. tuberculosis*) infection and the development of active tuberculosis. To evaluate whether *M. tuberculosis* infection susceptibility is associated with an intrinsic factor in monocytes from type 2 diabetes (T2D) patients or it is associated with hyperglycemia *per se*, we analyzed TLR-2 and TLR-4 expression by flow cytometry and the cytokines IL-1*β*, IL-6, IL-8, IL-10, and TNF-*α* by cytometric bead array assays, either stimulated with TLR-2 and TLR-4 ligands or infected with *M. tuberculosis* in the whole blood from T2D patients (*n* = 43) and healthy subjects (*n* = 26) or in CD14+ monocytes from healthy subjects cultured in high glucose (HG) (30 mM). The intracellular growth of *M. tuberculosis* was evaluated by CFU counts at 0, 1, and 3 days in both monocytes from T2D patients and monocytes from healthy subjects cultured in HG. We did not find significant differences in TLR expression, cytokine production, or growth of *M. tuberculosis* in monocytes from T2D patients compared with those in monocytes from healthy subjects. Despite these results, *in vitro* assays of monocytes cultured with 30 mM glucose led to significantly increased TLR-2 and TLR-4 basal expression compared to those of monocytes cultured with 11 mM glucose (*P* < 0.05). Conversely, the production of IL-6 by TLR-2 ligand stimulation, of IL-1*β*, IL-6, and IL-8 by TLR-4 ligand stimulation, and of IL-8 by *M. tuberculosis* infection significantly decreased in monocytes cultured in HG (*P* < 0.05). Additionally, the intracellular survival of *M. tuberculosis* increased in monocytes in HG after day 3 of culture (*P* < 0.05). In conclusion, HG decreased IL-8 production and the intracellular growth control of *M. tuberculosis* by monocytes, supporting the hypothesis that hyperglycemia plays an important role in the impaired immune responses to *M. tuberculosis* in patients with T2D.

## 1. Introduction

Diabetes mellitus (DM) represents a risk factor for *Mycobacterium tuberculosis* (*M. tuberculosis*) infection and the development of active tuberculosis (TB) [1]. Meta-analysis studies have shown that DM patients have an approximately 3-fold higher risk of developing TB disease [2], and TB control treatment is difficult in these patients [3]. The risk of developing active TB among diabetic patients is particularly high in Hispanic people, perhaps because latent TB infection is more common in these populations [4]. The frequency of DM among patients with pulmonary TB is 29.63%, and these patients have more severe clinical manifestations (i.e., a higher frequency of cavities on chest X-rays) and higher risks of recurrence and relapse than non-DM individuals with pulmonary TB [4], with poor glycemic control being a risk factor for TB [5].

DM is a metabolic disease characterized by hyperglycemia or high glucose (HG) concentrations in blood resulting from defects in insulin secretion, insulin action, or both. Hyperglycemia induces a chronic inflammatory state through the secretion of proinflammatory cytokines in type 2 diabetes (T2D) patients [2, 6]. It is noteworthy that in spite of the activation state characteristic in these individuals, they still possess a higher susceptibility to developing infections by many pathogens, including *M. tuberculosis* [2] than non-T2D individuals, which makes it difficult to dissect and identify the participant mechanisms associated with the predisposition to infections seen in diabetic patients. Nevertheless, hyperglycemia has been suggested as an important factor in *M. tuberculosis* infection susceptibility [6].

In physiological conditions, the mechanism of inflammation through the activation of monocytes and macrophages results after pathogen recognition by protein receptors, such as the toll-like receptor (TLR) family. HG concentrations are able to upregulate TLR-2 and TLR-4 and activate nuclear factor-*κ*B (NF-*κ*B) to induce proinflammatory cytokine production in THP1 monocytes in the absence of pathogens [7]. TLRs play a critical role in both innate immune responses and the initiation of adaptive immunity to *M. tuberculosis*, specifically TLR-2 and TLR-4. TLR activation upregulates the transcription of proinflammatory cytokines, including IL-1*β*, IL-6, and TNF-*α*, which are essential for the recruitment of immune cells to the site of infection and the control of *M. tuberculosis* infection [8].

In monocytes from T2D patients, TLR-2 and TLR-4 expression is increased [9, 10], and these patients present chronic low-grade inflammation with secretion of TNF-*α* and IL-8. The proinflammatory cytokines are induced by different mechanisms, such as TLR-2/4 activation (TLR-4 activation is induced by elevated exogenous ligands, such as dietary fatty acids and enteric lipopolysaccharide, and endogenous ligands, such as free fatty acids, which are elevated in obese states), reactive oxygen species (ROS) generation, or CD33 downregulation [9, 11–13]. Those mechanisms are important for pathogen control; however, the ROS and proinflammatory cytokines induced by HG do not protect against infections. In contrast, T2D patients are more susceptible than non-T2D individuals to infections, including *M. tuberculosis* infection, suggesting an immune susceptibility induced by HG concentrations.

The immunologic mechanisms of susceptibility to *M. tuberculosis* in T2D patients are still not fully understood. To investigate whether susceptibility to tuberculosis is due to an intrinsic alteration of monocytes from T2D patients, we used a whole-blood assay to preserve the physiological environment. To address whether the enhanced susceptibility to tuberculosis is due to HG concentrations *per se*, we used an *in vitro* model of hyperglycemia (using 30 mM glucose). In both assays, TLR-2 and TLR-4 expression, the cytokines released after TLR activation, and the growth control of *M. tuberculosis* were evaluated.

## 2. Materials and Methods

### 2.1. Study Population

Forty-three T2D patients were recruited from the Metabolic Syndrome Clinic of the National Institute of Respiratory Diseases in Mexico City according to the diagnosis criteria of the American Diabetes Association (Diagnosis and Classification of DM) [14]. Twenty-six healthy subjects (based on clinical laboratory tests) were invited to participate in this study. The Ethics Committee of the National Institute of Respiratory Diseases (INER) approved this study, and all participants provided written informed consent. Routine laboratory tests on blood as well as chest X-rays and Mantoux tuberculin skin tests (TSTs) were conducted (Table 1). Additionally, buffy coats from healthy adult donors were kindly provided by the blood banks at the INER.

### 2.2. High-Glucose Medium

Unless otherwise specified, all cells were cultured in RPMI 1640 medium according to formula specification with 11 mM glucose (Cambrex, Walkersville, MD, USA) that was supplemented with 50 *μ*g/ml gentamicin sulfate, 2 mM L-glutamine, and 10% heat-inactivated pooled human serum (Gemini Bioproducts, Sacramento, CA, USA) at 37°C in a 5% CO_2_ atmosphere. Culture medium with 30 mM (540 mg/dl) D-glucose (Sigma, St. Louis, MO, USA) is referred to in the text as HG.

### 2.3. Human Peripheral Monocyte Isolation

Sixty milliliters of heparinized human peripheral venous blood was obtained from the participants, and peripheral blood mononuclear cells were obtained from whole blood by centrifugation using Lymphoprep® (Nycomed Pharma, Oslo, Norway). Monocytes were positively selected by using MACS® magnetic beads coupled to an anti-human CD14 antibody (Miltenyi Biotec, Auburn, CA, USA) according to the manufacturer's recommendations. Purity was assessed by conventional flow cytometry; preparations exhibited >90% monocytes. Cell viability was assessed by trypan blue exclusion in all experiments and was >95%.

### 2.4. Mycobacterial Preparation

The *M. tuberculosis* strain H37Ra (25177, ATCC, Manassas, VA) was grown to a log phase in Middlebrook 7H9 broth (Difco Laboratories, Detroit, MI) supplemented with 1% glycerol and 10% albumin dextrose catalase enrichment (ADC, Becton Dickinson Co., MD) on an orbital shaking incubator at 37°C. Mycobacteria were harvested and frozen at -80°C until use (“stock bacteria”). For the infection assay, “stock bacteria” were thawed, and to remove any remaining large clumps, bacterial pellets were homogenized by passing them through a 26-gauge needle and centrifuged. The bacterial concentration was determined by serial dilution, and colony-forming units (CFU) were counted on Difco Middlebrook 7H10 agar plates (Becton Dickinson Co, MD) after disruption of mycobacterial clumps. *M. tuberculosis* was dyed with fluorescein diacetate (Fluka, Chemical, Switzerland) in 1 ml of phosphate-buffered saline (pH 9.1) for 30 min at 4°C.

### 2.5. TLR Surface Expression by Flow Cytometry

The whole blood from T2D and healthy subjects or CD14+ monocytes from healthy subjects cultivated *in vitro* with HG were stained with phycoerythrin- (PE-) labeled anti-human TLR-2, TLR-4, and CD14-FITC antibodies (eBioscience, San Diego, CA) or isotype controls. Cells were fixed with 1% paraformaldehyde, and TLR-2 and TLR-4 surface expression was analyzed in the monocyte region by flow cytometry. The results were analyzed as the percentage of positive cells and by mean fluorescence intensity (MFI) based on 10,000 events acquired in a FACSCanto II cytometer (BD). FlowJo software version 10.4.1 Mac OS X was used for analysis.

### 2.6. Cytokine Production in Response to TLR Ligands

One milliliter of whole blood from T2D and healthy subjects or 0.5 × 10^6^ CD14+ monocytes/well placed in 24-well plates were cultured in HG (30 mM) for 24 h and infected with *M. tuberculosis* (MOI of 10) or stimulated with 1 ng/ml TLR-2 ligand, synthetic lipoprotein palmitylated N-acyl-S-diacylglyceryl cysteine (Pam_3_Cys; EMC Microcollections, Tuebingen, Germany), or 100 ng/ml TLR-4 ligand, *Escherichia coli* lipopolysaccharide (LPS; Sigma), and were further incubated for 4 h for T2D and healthy subjects or 24 h for monocytes in HG. Supernatants were collected, and interleukin-1*β* (IL-1*β*), interleukin-6 (IL-6), interleukin-8 (IL-8), interleukin-10 (IL-10), and tumor necrosis factor-*α* (TNF-*α*) levels were measured by a cytometric bead array (BD Bioscience) following the manufacturer's recommendation. CellQuest software was used for sample acquisition, and data were formatted using BD Bioscience CBA software. The results were based on a standard concentration curve, and the cytokine concentration was expressed as pg/ml.

### 2.7. *M. tuberculosis* Growth Assay

CD14+ monocytes from T2D patients or healthy subjects were resuspended in RPMI 1640 medium, or monocytes from healthy subjects were resuspended in HG medium and cultured for 24 h. Then, 10^5^ monocytes/well were plated in 96-well flat plates (Corning Inc., Corning, NY) in triplicate and infected with *M. tuberculosis* H37Ra (MOI of 10) for 1 h. After removal of extracellular (nonphagocytosed) mycobacteria, RPMI or HG medium was added, and intracellular *M. tuberculosis* growth was determined. Briefly, after 0, 1, and 3 days of culture, cells were lysed with 0.1% sodium dodecyl sulfate (Sigma) for 10 min and then neutralized with 20% bovine serum albumin (Sigma). The lysates were serially diluted and plated in triplicate into Middlebrook 7H10 agar and incubated for 21 days at 37°C in 5% humidified CO_2_. The CFU were counted and reported as CFU/ml. In a set of control experiments, cell viability was assessed via a trypan blue exclusion assay (>90%) on days 0, 1, and 3 to estimate cell death as a result of treatment.

### 2.8. Statistical Analysis

Comparisons between T2D patients and healthy subjects or between RPMI and HG were made using the nonparametric Mann-Whitney rank sum test with GraphPad Prism 6.0h for Mac (GraphPad Software Inc.). The median with range is shown in the table, and the median is shown in all the figures. Statistical significance was set at *P* < 0.05.

## 3. Results

### 3.1. Patients and Healthy Subjects

The clinical and demographic characteristics of the T2D patients and healthy subjects are summarized in Table 1. No significant differences in sex, age, or levels of creatinine or HDL or LDL cholesterol between T2D patients and healthy subjects were observed; however, both groups were overweight. The levels of glucose, HbA1c, and triglycerides were significantly higher among T2D patients than among healthy subjects (*P* < 0.05).

### 3.2. The Expression of TLR-2 and TLR-4 in Monocytes from T2D Patients Is Similar to That in Monocytes from Healthy Subjects

**Figure 1:**
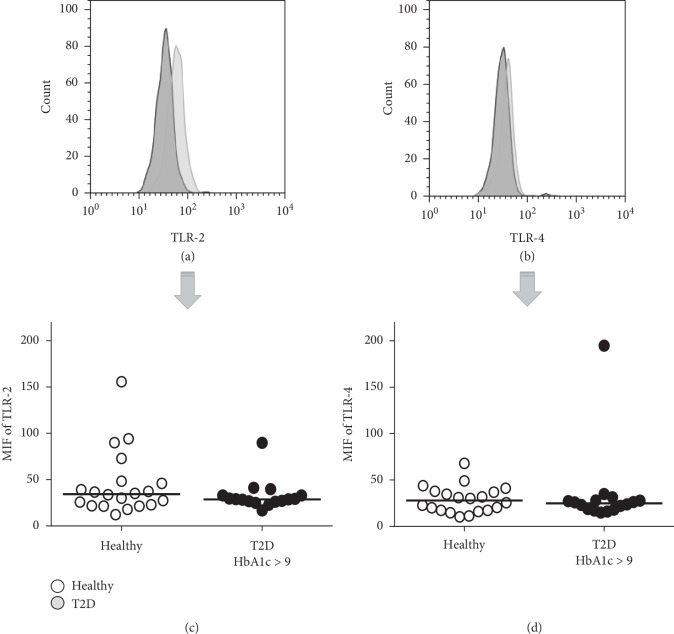
TLR cell surface expression in monocytes in T2D patients and healthy subjects. TLR surface expression was analyzed in whole blood samples by surface staining with AbM anti-human TLR-2PE or TLR-4PE and analyzed by flow cytometry. The monocyte region was analyzed, and TLR expression was reported as MFI. A representative experiment examining (a) TLR-2 and (b) TLR-4 in T2D and healthy subjects by cytometry is shown. Additionally, the surface expression of (c) TLR-2 and (d) TLR-4 in healthy subjects (open circle, *n* = 20) and T2D patients with HbA1c > 9 (closed circle, *n* = 16) is shown. The median is represented by a horizontal line.

TLRs are essential for pathogen recognition. To analyze the expression of TLR-2 and TLR-4 cell surface receptors in monocytes from T2D patients, we evaluated the TLR-2 and TLR-4 expression in the monocyte region in the whole blood from T2D patients by flow cytometry. We do not find any differences in the T2D group vs. healthy subjects; because of this, we analyzed a subgroup with an HbA1c of 9% and above (poor glycemic control). As shown in Figures 1(a) and 1(b), TLR expression in T2D and healthy subjects was assessed on monocytes, and the cell surface expression of TLR-2 (median, 28.7 for T2D and 34.3 for healthy) and TLR-4 (median, 24.8 for T2D and 27.9 for healthy) was similar in monocytes from T2D and healthy subjects (Figures 1(c) and 1(d)). The percentage of monocytes expressing TLR-2 and TLR-4 in healthy and T2D patients was similar in both groups (data not shown).

### 3.3. Cytokine Production in Response to TLR-2 and TLR-4 Activation or *M. tuberculosis* Infection Is Similar in T2D and in Healthy Subjects

**Figure 2:**
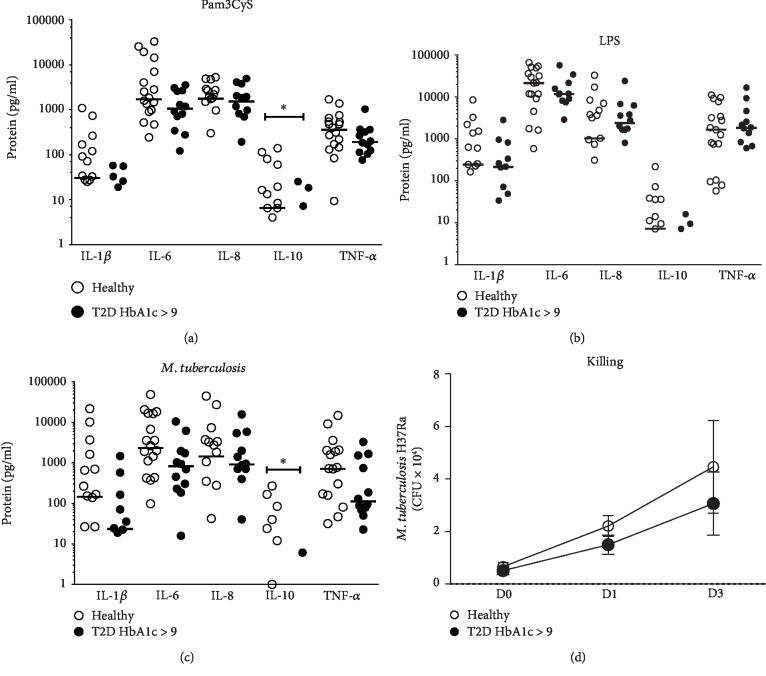
Cytokine production after TLR-specific stimulation or *M. tuberculosis* infection and intracellular growth control in monocytes from T2D patients and healthy subjects. Cytokine production elicited by activation through TLRs or *M. tuberculosis* infection was measured in the culture supernatants. Interleukin-1*β* (IL-1*β*), interleukin-6 (IL-6), interleukin-8 (IL-8), interleukin-10 (IL-10), and tumor necrosis factor alpha (TNF-*α*) protein levels were measured by a CBA-based assay and reported as pg/ml. Monocytes from healthy subjects (open circle, *n* = 17) and T2D patients with HbA1c > 9 (closed circle, *n* = 12) were stimulated with (a) Pam3Cys or (b) LPS or infected with (c) *M. tuberculosis*. Mann-Whitney *U* test, healthy subjects vs. T2D patients with HbA1c > 9. Monocytes from T2D patients and healthy subjects were infected with *M. tuberculosis* (MOI of 10) for 1 h; after the indicated time, cells were lysed with 1% SDS, and the mycobacteria were serially diluted and quantified in 7H10 agar media after 21 incubation days, which was reported as CFU. (d) *M. tuberculosis* growth in healthy subjects (open circle, *n* = 20) and T2D patients with HbA1c > 9 (closed circle, *n* = 19). The median is represented by a horizontal line.

To analyze TLR activation in monocytes from T2D patients and healthy subjects, further experiments were conducted to evaluate the IL-1*β*, IL-6, IL-8, IL-10, and TNF-*α* levels induced by TLR ligands, Pam3Cys or LPS, or *M. tuberculosis* infection in monocytes from T2D patients with an HbA1c of 9% and above (poor glycemic control) and healthy subjects. Cytokine production was determined after stimulation with Pam3Cys (median (pg/ml), IL-1*β*—0 and 32.9, IL-6—1058.9 and 1600.7, IL-8—1529.2 and 1740.6, and TNF-*α*—193.6 and 402.1 for T2D and healthy, respectively), LPS (median (pg/ml), IL-1*β*—213.4 and 246.0, IL-6—11762.8 and 21460.7, IL-8—2382.5 and 1030.6, IL-10—0 and 7.2, and TNF-*α*—1830.6 and 1660.9 for T2D and healthy, respectively), and *M. tuberculosis* infection (median (pg/ml), IL-1*β*—23.3 and 153.0, IL-6—827.1 and 2673.6, and IL-8—913.3 and 1832.8 for T2D and healthy, respectively). Lower IL-10 production was observed in T2D only after Pam3Cys stimulation (median (pg/ml), 0.0 (0-25.3) in T2D and 6.5 (0-139.5) in healthy subjects) and after *M. tuberculosis* infection (median (pg/ml), 0.0 (0-6.1) in T2D and 0 (0-270) in healthy subjects) (*P* < 0.05, Figures 2(a)–2(c)). We analyzed the correlation of cytokines with BMI; for T2D patients, no correlation was found (*P* > 0.05), and for healthy subjects, just TNF-*α* and BMI correlate weakly after LPS stimulation (*P* > 0.042, *r* = 0.535) ([Supplementary-material supplementary-material-1]).

### 3.4. The *M. tuberculosis* Growth in Monocytes from T2D Patients Is Similar to That in Monocytes from Healthy Subjects

Since T2D patients with poor glycemic control show increased susceptibility to *M. tuberculosis* infection, we evaluated the capability of monocytes from T2D patients to control *M. tuberculosis* intracellular growth. Monocytes were cultured in RPMI medium for 3 days, and the CFU of *M. tuberculosis* were counted on 7H10 agar plates. As shown in Figure 2(d), the intracellular growth of *M. tuberculosis* in monocytes from T2D patients was similar to that in monocytes from healthy subjects (median, 3.8 × 10^3^—D0, 3.0 × 10^4^—D1, and 2.3 × 10^4^—D3 for healthy subjects and 3.9 × 10^3^—D0, 2.5 × 10^4^—D1, and 2.1 × 10^4^—D3 for T2D patients).

### 3.5. High Glucose Concentrations Upregulate TLR-2 and TLR-4 Expression in Monocytes from Healthy Subjects

**Figure 3:**
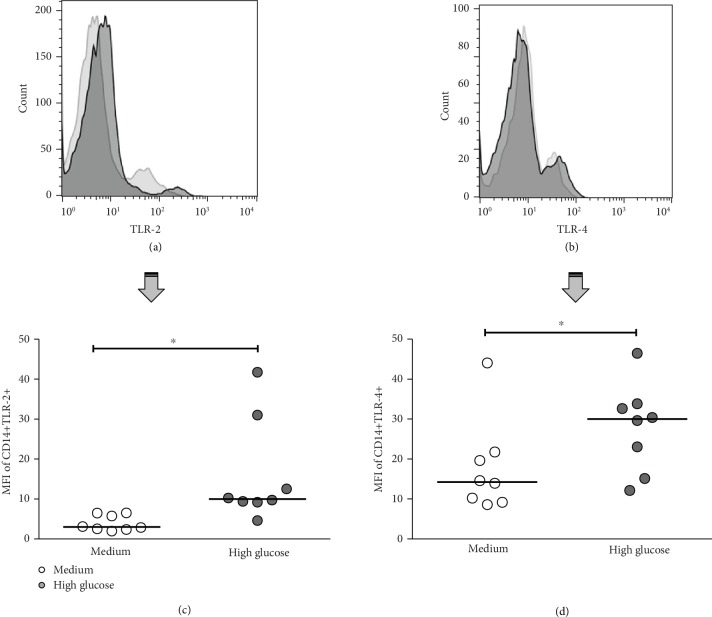
Effect of high glucose on TLR-2 and TLR-4 surface expression in human monocytes from healthy subjects. Monocytes were cultured in high glucose (30 mM) or RPMI medium for 24 h and then stained with specific monoclonal antibodies against human CD14, and the TLR and monocyte regions were analyzed. A representative analysis of the effect of HG on (a) TLR-2 and (b) TLR-4 is shown. Expression of (c) TLR-2 and (d) TLR-4 in monocytes cultured in RPMI medium (closed circles) or under HG (30 mM) (gray circles) (*n* = 8). Mann-Whitney *U* test, ^∗^*P* < 0.05, medium vs. HG. The median is represented by a horizontal line.

Because we observed that the TLR expression in T2D patients was not affected, for a next hypothesis, we evaluated whether the glucose *per se* affects the main innate immune mechanisms for *M. tuberculosis* recognition. We analyzed TLR cell surface expression using an *in vitro* model of HG (30 mM or 540 mg/dl glucose). For this experiment, monocytes were cultured in HG for 24 h, and TLR-2 and TLR-4 expression was subsequently determined by flow cytometry. As shown in Figures 3(a)–3(d), both TLR-2 (median, RPMI medium 15.35 and HG 51.28) and TLR-4 (median, RPMI medium 14.27 and HG 30.03) cell surface expression was upregulated significantly in monocytes cultured in HG compared to cells cultured with RPMI medium (*P* < 0.05).

### 3.6. Elevated Glucose Concentrations Decrease Cytokine Production in Response to TLR-2 and TLR-4 Ligands or *M. tuberculosis* Infection

**Figure 4:**
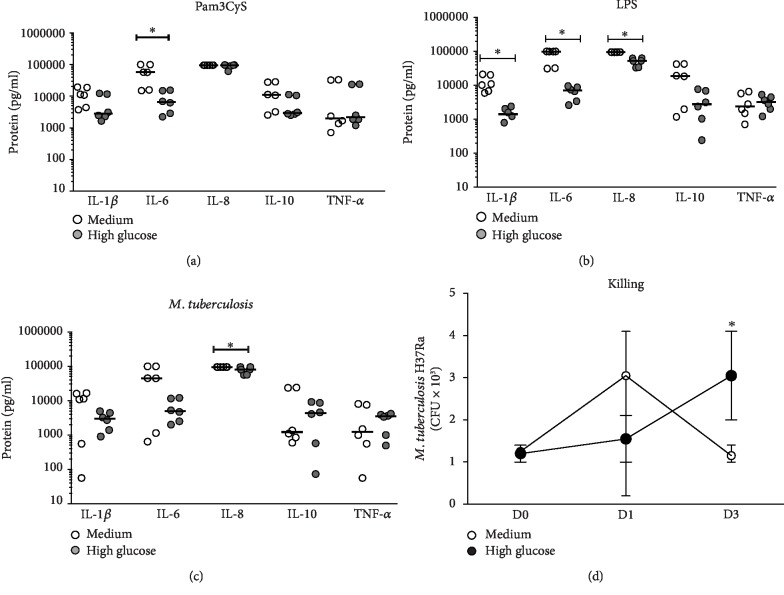
Effect of high glucose on cytokine production after TLR-specific stimulation or *M. tuberculosis* infection and intracellular growth control of the mycobacteria in human monocytes. Monocytes were infected with *M. tuberculosis* (MOI of 10) for 1 h. Subsequently, cells were transferred to RPMI medium or HG (30 mM) and cultured for 0, 1, and 3 days. After the indicated time, the supernatants were analyzed for IL-1*β*, IL-6, IL-8, IL-10, and TNF-*α* cytokines, as determined by the CBA-based assay. The cells were lysed with 1% SDS, and the mycobacteria were serially diluted and plated on 7H10 agar media. After 21 days of incubation, CFU were determined. Cytokine production after (a) P3Cys TLR-2 ligand and (b) LPS TLR-4 ligand stimulation or (c) *M. tuberculosis* infection in culture supernatants in RPMI medium (open circles) and HG (gray circles) (*n* = 6). The growth control of (d) *M. tuberculosis* in RPMI medium (open circles) and HG (closed circles) is shown (*n* = 6). Mann-Whitney *U* test, ^∗^*P* < 0.05, RPMI medium vs. HG. The median is represented by a horizontal line.

To identify the effect of HG concentration on TLR-specific activation of monocytes, we evaluated cytokine production in monocytes cultured in HG for 24 h and after stimulation with Pam3Cys or LPS as TLR-2- or TLR-4-specific ligands, respectively, or *M. tuberculosis* infection for 24 h. HG decreased IL-6 production through TLR-2 activation (median, RPMI medium 57085 pg/ml and HG 6521 pg/ml, *P* < 0.05, Figure 4(a)), while the production of cytokines through TLR-4 activation also decreased; IL-1*β* (median, RPMI medium 10608 pg/ml and HG 1418 pg/ml), IL-6 (median, RPMI medium 99400 pg/ml and HG 7147 pg/ml), and IL-8 (median, RPMI medium 95400 pg/ml and HG 53115 pg/ml) levels all were decreased (*P* < 0.05, Figure 4(b)). Additionally, HG decreased IL-8 production in response to *M. tuberculosis* infection in HG compared to RPMI medium (median, RPMI medium 95250 pg/ml and HG 80346 pg/ml, *P* < 0.05, Figure 4(c)).

### 3.7. High Glucose Reduces Intracellular *M. tuberculosis* Growth in Monocytes

After phagocytosis, macrophages induce antibacterial mechanisms, including cytokine production, that inhibit the intracellular growth of *M. tuberculosis* in monocytes. We evaluated the intracellular *M. tuberculosis* growth in monocytes cultured in HG.  Figure 4(d) shows an increase in intracellular *M. tuberculosis* growth in monocytes after 3 days of culture with HG with respect to that in monocytes with RPMI medium (*P* < 0.05) (median, RPMI medium: 1.2 × 10^3^—D0, 1.7 × 10^3^—D1, and 3.0 × 10^3^—D3 and HG: 1.2 × 10^3^—D0, 3.4 × 10^3^—D1, and 1.1 × 10^3^—D3).

## 4. Discussion

T2D is a metabolic disease with high levels of glucose in the blood and an increased risk of developing active TB. To address the immune mechanisms associated with the *M. tuberculosis* response in T2D patients and since TLR-2 and TLR-4 play critical roles in TB infection, we evaluated the TLR expression, proinflammatory cytokine production, and intracellular growth of *M. tuberculosis* in monocytes from T2D patients to assess the intrinsic impairment of monocytes in *M. tuberculosis* recognition and control. We also used an *in vitro* hyperglycemia model (30 mM glucose) to assess whether high glucose impaired the control of *M. tuberculosis*. In this population, we did not find a significant difference in TLR-2 and TLR-4 expression, cytokine production, or intracellular growth of *M. tuberculosis* in T2D patients compared to healthy subjects. However, using the *in vitro* model of hyperglycemia at 30 mM glucose, we found that cytokine production was diminished after TLR-2 and TLR-4 activation and that the intracellular growth of *M. tuberculosis* in human monocytes was not controlled.

TLRs are an important mechanism for the recognition of *M. tuberculosis*; TLR-2 and TLR-4 play critical roles in TB infection and are the immediate mechanism for the recognition of pathogens [15, 16]. Also, TLRs are important regulators of the immune and metabolic systems, including in DM [17]. However, the role of TLRs in diabetes is controversial; these apparent discrepancies could be explained by the population, experimental design, and type of cell. Dasu et al. report an increased TLR-2 and TLR-4 expression in monocytes from recently diagnosed type 2 diabetic patients [9]. Ahmad et al. report an increased expression of TLR-2 and TLR-4 in PBMCs from obese and overweight individuals with type 2 diabetes [18]. Gupta et al. report no differences in TLR-2, TLR-4, and TLR-6 levels in monocytes from patients with good glycemic control when compared to nondiabetic volunteers [19]. In addition, it was reported that the expression of TLRs did not differ significantly between T2D patients and healthy voluntaries, although their monocytes are less responsive to TLR ligands [20]. Using the whole blood assay to preserve physiological environment and avoid possible artificial stimulation of monocytes, Khondkaryan et al. evaluate the expression of TLR-2 and TLR-4 on the total monocyte surface in culture from the whole blood and report significantly reduced TLR-4 expression in unstimulated T2DM monocytes compared to respective cells from healthy donors [21]. In concordance with Khondkaryan et al., we observed that T2D patients express TLR-2 and TLR-4 in monocytes at the same level as healthy subjects; although the healthy population was overweight, a previous report demonstrated that subjects with T2D had significantly elevated mRNA levels of TLR-2 and TLR-4 compared with nondiabetic obese subjects [18]. These contradictory results show that the levels of TLR-2 and TLR-4 are not a hallmark of T2D patients and the levels of TLR expression are rather associated with factors such as glucose levels.

Additionally, in *in vitro* studies, HG increased the expression of TLR-2 and TLR-4 in THP1 cells and primary monocytes from healthy subjects via protein kinase C (PKC) [7]. We also observed the *in vitro* effect of HG on the upregulated expression of TLR-2 and TLR-4 on the cell surface of primary monocytes from healthy subjects, similar to recently diagnosed type 2 diabetic patients [9], supporting the upregulation of TLRs in the presence of HG concentrations.

After pathogen recognition, TLRs activate signaling cascades via NF-*κ*B to induce proinflammatory responses. The role of hyperglycemia as a proinflammatory factor was demonstrated by *in vitro* models. In THP-1 monocytes, HG concentrations (15 mM) induce the production of TNF-*α*, interleukin- (IL-) 1*β*, chemokines, such as monocyte chemoattractant protein-1 (MCP-1), and interferon gamma inducible protein-10 (IP-10), many of which are NF-*κ*B regulated [22]. Previous reports on models of diabetic rats infected with *M. tuberculosis* showed a reduced production of TNF-*α*, IFN-*γ*, and IL-12 by alveolar macrophages [23]. In contrast, in patients with diabetes who had poorly controlled blood glucose levels, TNF-*α*, IFN-*γ*, IL-2, and IL-1*β* cytokine levels were increased in response to PPD [24]. However, the response was different when we used the live *M. tuberculosis* H37Ra strain. We observed similar levels of TNF-*α*, IL-1*β*, IL-6, and IL-8 cytokine production, whereas in response to Pam3Cys, a synthetic ligand of TLR-2, only IL-10 production was reduced in T2D patients. A similar response was reported in diabetic mice, with low levels of IL-10 expression in macrophages from diabetic mice [25]. Additionally, HG decreases the release of IL-1*β*, IL-6, and IL-8 in LPS-stimulated monocytes cultured in the presence of HG. These results are partially consistent with Beitland et al. who found that IL-1*β* production was significantly reduced in LPS-stimulated blood samples in the presence of 20 mmol/l glucose [26]. However, the TLR responses differ in the type and magnitude of the innate immune responses they elicit when interacting with different ligands. We used live *M. tuberculosis* to evaluate the response by monocytes in HG and observed that IL-8 production was significantly reduced. Interleukin-8 (IL-8) displays two major biological activities: chemoattraction and activation of several types of white blood cells. IL-8 plays a central role in the normal immune response to *M. tuberculosis* and has been shown to be absolutely required for granuloma formation. Monocytes and macrophages infected with *M. tuberculosis* are the primary producers of IL-8 during the course of TB disease, an inability to stimulate the production of IL-8 correlated with a poor prognosis in patients with TB [27], and the reduction in IL-8 production by HG could participate in tuberculosis susceptibility.

It has been well established that recognition and phagocytosis of *M. tuberculosis* induce the production of antimicrobial peptides, which are important for the control of the intracellular growth of pathogens, including *M. tuberculosis* [28]. We observed that the growth of *M. tuberculosis* increased in monocytes cultured in HG concentrations. The reduced cytokine production observed in this study suggests inadequate activation through these receptors, which may be a putative mechanism of the reduction in TLR-mediated *M. tuberculosis* control. These findings are consistent with previous studies in animal models of diabetes, in which mice infected with *M. tuberculosis* did not control the infection and died in a short time [29]. Additionally, diabetic rats (with HG levels in their urine) had a large number of granulomas, with a high *Mycobacterium* load [23]. Our study presents evidence of the role of hyperglycemia in the mechanism associated with *M. tuberculosis* intracellular control.

## 5. Conclusion

TLR-2 and TLR-4 expression, cytokine production, and the intracellular growth of *M. tuberculosis* are not associated with an intrinsic factor in monocytes from T2D patients; however, the presence of high glucose concentrations decreased cytokine production and increased the intracellular growth of *M. tuberculosis* in monocytes, suggesting that hyperglycemia at high levels may play a key role in altering the immune responses to *M. tuberculosis* in diabetic patients.

## Figures and Tables

**Table 1:** The clinical and demographic characteristics of the T2D patients and healthy subjects.

	Healthy (*n* = 26)	T2D (*n* = 43)	^∗^ *P* ≤ 0.05
Sex (male/female)	9/17	15/28	ns
Age (years)	48 [33-65]	53 [30-68]	ns
Weight (kg)	65 [51-95]	66 [51-114]	ns
Height (m)	1.58 [1.45-1.80]	1.56 [1.44-1.74]	ns
BMI (kg/m^2^)	26 [23-33]	26 [23-47]	ns
Fasting glucose (mg/dl)	99 [80-115]	210 [106-460]	^∗^
HbA1c (%)	5.5 [5.0-5.9]	8.9 [6.1-15.6]	^∗^
HbA1c > 9 (*n* = 19)	—	10.3 [9.4-15.6]	
Creatinine (mg/dl)	0.77 [0.59-1.10]	0.71 [0.52-1.84]	ns
Cholesterol (mg/dl)	186 [136-313]	200 [147-310]	ns
Triglycerides	132 [70-469]	215 [46-688]	^∗^
HDL (mg/dl)	39 [26-67]	43.2 [29-70]	ns
LDL (mg/dl)	124 [76-208]	125 [11-228]	ns
Status TST (+/-)	47.8% (11/11)	57.5% (23/17)	ns

T2D = type 2 diabetes mellitus, BMI=body mass index, HbA1c = glycated haemoglobin, HDL = high-density lipid, LDL = low-density lipid, TST = tuberculin skin test, ns = not significant. Values are median [Min–Max]. *t*-test = T2D vs. healthy. Two-sample proportion test for sex and TST.

## Data Availability

The research data used during this study are included in the paper. The clinical and demographic data for each patient or healthy subject analyzed in this study are available from the corresponding author upon request.

## Supplementary Materials

Supplementary Materials Correlation of cytokine production and BMI in T2D patients and healthy subjects. The whole blood from T2D (HbA1c >9%) and healthy subjects was stimulated with Pam3Cys (TLR-2 ligand) or LPS (TLR-4 ligand) or infected with *M. tuberculosis* (MOI of 10) and incubated for 4 h. Supernatants were collected, and IL-1*β*, IL-6, IL-8, IL-10, and TNF-*α* levels were measured by a cytometric bead array, and the cytokine concentration was reported as pg/ml. Correlation was done between cytokine concentration and body mass index (BMI) using the Spearman test. The dot graphics represent (a) T2D patients (close circle, *n* = 13) and (b) healthy subjects (open circle).
