# Supplementary figures and images for: The Intracellular Growth of M. tuberculosis Is More Associated with High Glucose Levels Than with Impaired Responses of Monocytes from T2D Patients
Source: J Immunol Res. 2019 Nov 14;2019:1462098. doi: 10.1155/2019/1462098 (PMC6877949; doi:10.1155/2019/1462098)

## Slide 1
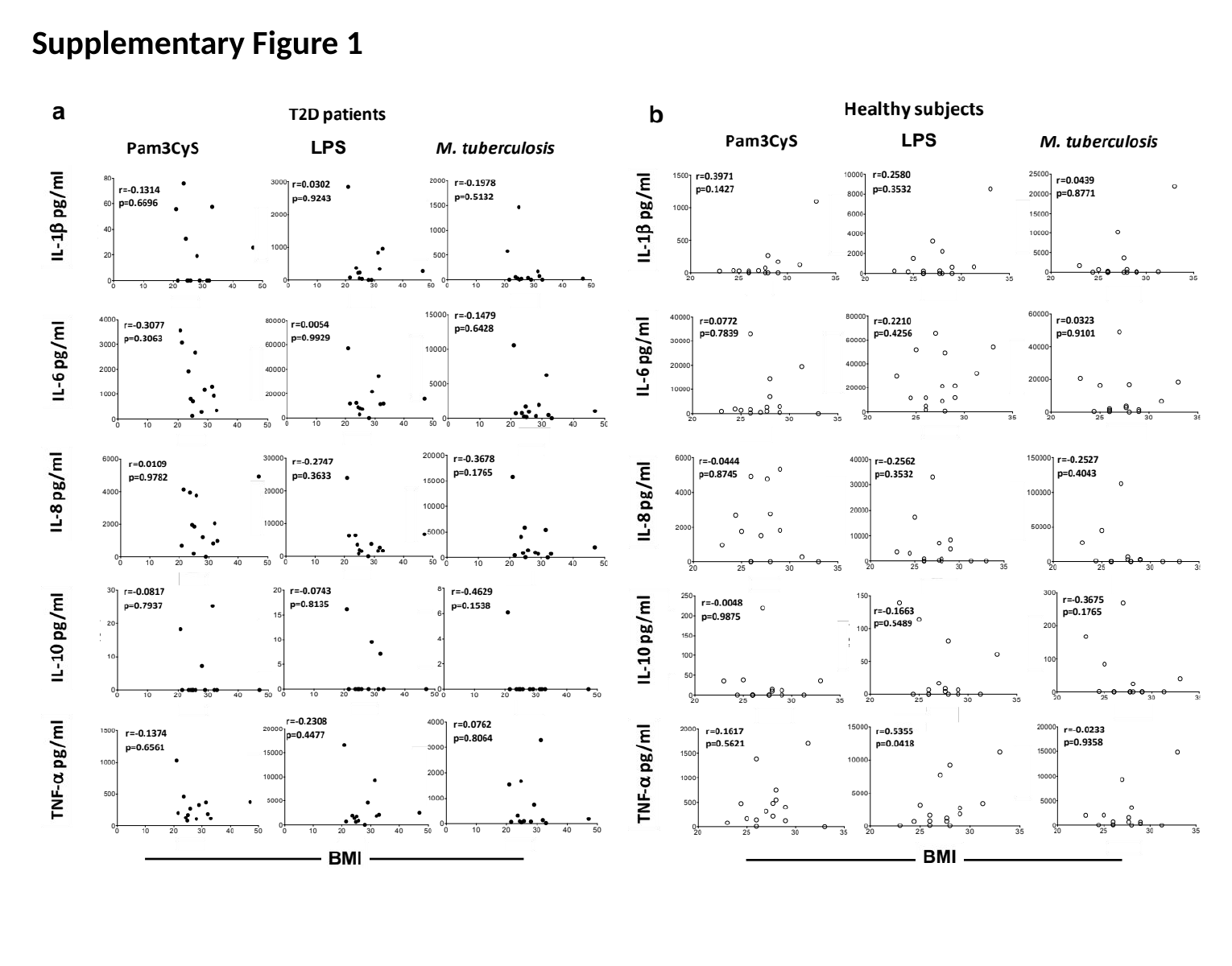

Supplementary Figure 1

Supplement: Supplementary Materials — Correlation of cytokine production and BMI in T2D patients and healthy subjects. The whole blood from T2D (HbA1c >9%) and healthy subjects was stimulated with Pam3Cys (TLR-2 ligand) or LPS (TLR-4 ligand) or infected with M. tuberculosis (MOI of 10) and incubated for 4 h. Supernatants were collected, and IL-1β, IL-6, IL-8, IL-10, and TNF-α levels were measured by a cytometric bead array, and the cytokine concentration was reported as pg/ml. Correlation was done between cytokine concentration and body mass index (BMI) using the Spearman test. The dot graphics represent (a) T2D patients (close circle, n = 13) and (b) healthy subjects (open circle). [file 1462098.f1.pptx]
